# Protocol for a nationwide case-control study of firearm violence prevention tactics and policies in K-12 schools

**DOI:** 10.1371/journal.pone.0302622

**Published:** 2024-05-20

**Authors:** Navjot Buttar, Sonali Rajan, Louis Klarevas, Seth J. Prins, Justin Heinze, Ken Cheung, Kara E. Rudolph, Monika Goyal, April Zeoli, Charles C. Branas

**Affiliations:** 1 Department of Epidemiology, Mailman School of Public Health, Columbia University, New York, NY, United States of America; 2 Department of Health Studies & Applied Educational Psychology, Teachers College, Columbia University, New York, NY, United States of America; 3 Department of Mathematics, Science and Technology, Teachers College, Columbia University, New York, NY, United States of America; 4 Department of Sociomedical Sciences, Mailman School of Public Health, Columbia University, New York, NY, United States of America; 5 Department of Health Behavior and Health Education, School of Public Health, University of Michigan, Ann Arbor, MI, United States of America; 6 Department of Biostatistics, Mailman School of Public Health, Columbia University, New York, NY, United States of America; 7 Children’s National Hospital, Washington, DC, United States of America; 8 Department of Health Management and Policy, School of Public Health, University of Michigan, Ann Arbor, MI, United States of America; 9 Institute for Firearm Injury Prevention, University of Michigan, Ann Arbor, MI, United States of America; PLOS: Public Library of Science, UNITED KINGDOM

## Abstract

**Background:**

Most U.S. K-12 schools have adopted safety tactics and policies like arming teachers and installing metal detectors, to address intentional school gun violence. However, there is minimal research on their effectiveness. Furthermore, sociodemographic factors may influence their implementation. Controlled studies are necessary to investigate their impact on gun violence and related disciplinary outcomes.

**Objective:**

The paper outlines the protocol for a case-control study examining gun violence prevention policies in U.S. K-12 schools. The study aims to investigate if there is an association between the total number and type of specific safety tactics and policies and the occurrence of intentional shootings in K-12 public schools, student disciplinary outcomes, and if urbanicity, economic, and racial factors modify these associations.

**Methods:**

We will create a nationally representative dataset for this study and ascertain a full census of case schools (schools that experienced intentional gunfire on the campus during school hours since 2015) through national school shooting databases. Matched control schools will be randomly selected from U.S. Department of Education’s national database of all public schools. We will analyze 27 school safety strategies organized into seven key exposure groupings.

**Results:**

Supported by the National Institutes for Child Health and Development (R01HD108027-01) and having received Institutional Review Board approval, our study is currently in the data collection phase. Our analytical plan will determine the association between the number and type of school safety tactics and policies with the occurrence of intentional shootings and suspensions and expulsions in a national sample of approximately 650 K-12 public schools. Additional analyses will investigate the effect modification of specific covariates.

**Conclusion:**

As the first national, controlled study, its results will provide novel and needed data on the effectiveness of school safety tactics and policies in preventing intentional shootings at K-12 public schools.

## Introduction

Gun violence is currently the leading cause of death among children and teens in the US [1,2]. Gun violence in K-12 public schools around the US persists as a public health crisis. Over 357,000 students have been exposed to gun violence in K–12 schools since the Columbine High School shooting in Colorado in 1999. These numbers do not fully capture the impact of school shootings on children, teachers, and other school staff who may not have been directly injured by firearms but may have experienced indirect forms of gun violence such as witnessing a shooting. The nature of this kind of violence significantly impacts the well-being of entire school communities, with enormous consequences for children’s health, learning, and development [3,4].

In response to the persistence of this kind of violence, K-12 schools across the US are widely implementing numerous safety tactics and policies as a way to secure their buildings and prepare their communities for the possibility of gunfire on school grounds. However, the effectiveness of these security tactics and policies at deterring school shootings remains largely untested. Implementation of security measures, such as metal detectors, armed school staff, and zero-tolerance policies has significantly changed the daily school experience since Columbine [5]. In 1999, less than 20% of schools had security cameras; now more than 80% do [6]. Similarly, as of 2019, 96% of K-12 schools now conduct lockdown drills, compared with far fewer who implemented such procedures prior to the Sandy Hook school shooting in 2012 [7]. This is significant, as there are currently over 90,000 public K-12 schools across the nation, serving an estimated 51 million children. Unfortunately, there is limited and conflicting evidence surrounding these tactics and policies. Some studies show that more security measures in schools, such as metal detectors and armed guards, can result in students feeling less safe [8–10]. However, other work suggests that students feel safer with these policies [11]. Further, there is evidence that security measures have little effect [12]. Overall, studies evaluating school security policies have been inconclusive and inadequately designed with no comparator or control schools or student populations [13,14].

Thousands of instances of gunfire have occurred in US K–12 schools over the twenty years since the Columbine High School shooting [15]. However, following the Columbine shooting in 1999, the yearly incidence of school shootings remained relatively constant until a noticeable uptick beginning in 2015 [16]. Despite this recent increase, little comparative research has examined what specific safety tactics and policies work to prevent intentional shootings on K-12 school grounds [17]. Definitions of what counts as a “school shooting” currently range from an accidental discharge of a gun at school to injury or death of a student by a firearm. Although the prevention of accidental shootings or suicides are extremely important, our focus in this proposed study is on efforts aimed at the prevention of intentional shootings of other individuals in K-12 public schools, particularly since many existing school safety strategies have been designed specifically to prevent intentional, interpersonal gun violence.

### K-12 school security

In response to the anticipation of school gun violence, millions of K-12 students now walk through airport-style security every day in the US [17]. The effectiveness of such measures is understudied, but some research suggests that visible security measures, such as metal detectors, cameras, and police officers, do not improve school safety or academic achievement and may increase student anxiety and stress [18]. For instance, metal detectors cannot distinguish between different objects made of metal and often require searches by security personnel questioning their effectiveness, while possibly heightening student anxiety [19,20]. Security measures may also exacerbate disparities in school discipline outcomes. Available research on active shooters in schools has been conducted via small sample case-series studies. While limited, this research suggests that commonalities exist across individuals who commit school shootings, such as having experiences with bullying, access to weapons, and poor social skills [21,22]. Research has also illustrated that school shooters are typically teenage, middle-class, White males, that live in suburbs and rural neighborhoods [23] and with parents that own firearms [24].

Despite these known demographics, “hardening” of schools through addition of armed guards, metal detectors, and zero-tolerance policies is more likely to occur in schools that are primarily serving minoritized students and in urban areas of lower socioeconomic status, even after accounting for violent crime rates [3,4,6]. Thus, schools appear to differentially employ security interventions and disciplinary action based on sociodemographic factors unrelated to school safety [25–27]. Critics have argued that this criminalizes students for minor misbehaviors, possibly provoking pathways into the criminal justice system, a phenomenon often described as the school-to-prison pipeline [28–30]. Some patterns of school security usage are associated with increased exposure to crime and violence at school [31,32] and misusage of security may have detrimental effects on academic outcomes [33]. Little research has been completed using readily available data on suspensions and expulsions, as indicators of student criminalization, across disparate school types. In this way, school securitization is part of a broader, poorly understood policy trend to manage, rather than repair, consequences of disinvestment, economic austerity, and unprecedented social inequality. Research on the effectiveness and unintended negative consequences of school safety interventions in a wide variety of communities is critical.

### Prior methodological limitations

Prior studies of school safety tactics and school shootings have been case-series designs, i.e., only involving schools that have experienced shootings. This is a critical methodological shortcoming because there is no way to determine if the safety tactics and policies in question are protective with no comparison group of schools that have not experienced shootings (but that could have) and may have had many of the same safety tactics and policies in place at similar points in time. The present study is therefore employing a population-based, incidence density sampled case-control study of shootings in K–12 schools that includes a national group of schools that have experienced shootings and national comparator group of control schools that have not experienced a shooting.

### Purpose

Using this case-control study design, the study protocol has the following three specific aims and hypotheses,

- **Aim 1:** To determine if the total number and specific types of safety tactics and policies are associated with the occurrence of intentional shootings in K-12 public schools.
  ○ Primary Aim 1 hypothesis—The total number of cumulative safety tactics and policies will be significantly associated with intentional school shootings.
  ○ Secondary Aim 1 hypothesis—When organized into three domains (physical target hardening, emergency response and technologies, and school security), the total number of safety tactics and policies within each domain will be significantly associated with intentional school shootings.
- **Aim 2:** To determine if the total number and specific types of safety tactics and policies are associated with suspension and expulsion rates in K-12 public schools.
  ○ Primary Aim 2 hypothesis—The total number of cumulative safety tactics and policies will be significantly associated with student discipline outcomes.
  ○ Secondary Aim 2 hypothesis—When organized into three domains (physical target hardening, emergency response and technologies, and school security), the total number of safety tactics and policies within each domain will be significantly associated with student discipline outcomes.
- **Aim 3:** To identify if urban/non-urban, economic, and racial disparities modify the relationships between the implementation of safety tactics and policies, suspensions and expulsions, and intentional shootings in K-12 public schools.
  ○ Aim 3 Hypothesis: Significant urban/non-urban, economic, and racial disparities will modify the relationships between the implementation of safety tactics and policies, suspensions and expulsions, and intentional shootings in K-12 public schools.

## Methodology

### Sampling frame

National data from a random sample of K-12 public schools around the US are being collected for this case-control study. Case schools are being ascertained through a comprehensive review of multiple national school shooting databases, including the K-12 School Shooting database, the Everytown for Gun Safety database on gunfire on school grounds, and the Washington Post database on school shootings, to identify all schools that have experienced an intentional gunfire against others on the campus during school hours. Control schools will be ascertained via random selection from the US department of Education’s national database on all K-12 schools and matched to case schools in 1:1 ratio. The full set of inclusion and exclusion criteria developed by the study team are included [S1 File].

#### Case school ascertainment

We are primarily identifying all school shooting cases via the K-12 School Shooting database (SSDB), as these data detail “all incidents in which a gun is fired, or a bullet hits school property for any reason, regardless of the number of victims, time, day of the week” [34]. Two members of the research team have independently coded this list of incidents to identify the cases that meet the proposed study’s criteria for “intentional school shooting.” And a third member of the study team has reviewed any potential discrepancies in coding efforts. By the study’s completion, the list of cases will be a full, national census of schools that have experienced an intentional shooting on school property, during school hours (including one hour prior to official school activities starting and one hour after all official school activities end) beginning January 1, 2015. The study will exclude accidental discharges and attempted suicides where no other person was targeted or shot. It will also exclude any incidents taking place on a school bus or on school property that is non-contiguous with the school’s primary campus. This list of intentional school shootings meeting the study criteria will be cross-referenced via secondary inspection and inclusion of case data from a series of additional databases that also record and publicly report school shooting incidents, including the Washington Post school shooting incident database and the Everytown for Gun Safety list of gunfire on school ground. These databases are being linked and harmonized to detail all school shooting incidents by the definition above, from January 1, 2015 –December 31, 2023.

#### Control school ascertainment

Control schools are a randomly selected national sample of schools that have not experienced a shooting, by the definition described above and within the same time frame (1/1/2015–12/31/2023). Control schools are being obtained via random selection from a national database of public K-12 schools via the U.S. Department of Education’s National Center for Education Statistics (NCES). For each case school, one control school is being chosen as part of a stratified random sample and matched to each case school based on their geographic state, urban/non-urban status, and elementary/middle/high school status. State and urban/non-urban status will be determined using each school’s address and standard definitions of urbanicity taken from the NCES locale codes.

### Data collection

Driven by preliminary study planning and statistical power calculations, the study protocol intends to collect data on an estimated 658 K–12 public schools (329 cases and 329 controls) across the US.

#### Independent variable—school safety strategies

Data on school safety tactics and policies will be predominantly based on those listed in the School Survey on Crime and Safety (SSOCS); a national survey that is administered via the US Department of Education’s NCES to a representative sample of public schools every two years to assess a range of school safety and security interventions, among other indicators of school violence and school violence prevention. Drawing on this survey and also on the existing literature, our study team has identified a comprehensive set of 27 different school safety tactics and policies, which have been further grouped into seven categories (S2 File). Each school’s safety plan, their student handbook, their code of conduct, their school improvement plan, and/or the school’s facilities information, satellite and Google Street View images, and additional publicly available secondary data sources will be used to identify the specific security tactics and policies in place at both case and control schools during the school year before each pair-matched case school’s shooting event.

It should be noted that public school safety plans are comprehensive documents developed by school leaders that detail preparation and response protocols for a range of possible school situations and emergencies included in the proposed study. Data on school safety strategies are being obtained from each school’s publicly available written school safety plan or handbook online at local school district websites. In the case that a given school’s safety plan is not available online, the research team is emailing the superintendents of the school district directly to inquire if they are able to provide a copy of that specific school’s safety plan for the purposes of this study and in line with the study’s IRB protocol.

Furthermore, in completing the specific aims that address the impact of school safety tactics and policies at different points in time, we are obtaining school safety plans with valid timestamps of the years they went into effect, including the year of a case school shooting. To obtain complete safety information for each school in the study, researchers are triangulating the data obtained via the school safety plans with other reliable and date-stamped sources of data. Using the Google Street View “time machine” feature to obtain images of schools in the index year of each shooting, street-level and satellite imagery data are also being audited to confirm whether external structures of schools have illuminated school entry doors, external barriers, bollards or buffer zones, and/or external security cameras.

#### Publicly available school suspensions and expulsions data

Using Local Education Agency codes for each case and control school obtained from the NCES, annual data on suspensions and expulsions, percentage of discipline outcomes tied directly to zero-tolerance policies, and the frequency of interactions between schools and local law enforcement will be directly downloaded from the Civil Rights Data Collection Website where data is available for all K-12 schools [35]. These data for each case and control school will be linked and used to comprise a “school discipline” summative score that includes the following indicators (accounting for school population size): number of in-school suspensions, out-of-school suspensions, and student expulsions. Other data (e.g., the number of students referred to law enforcement and number of school-related arrests) will also be collected from the Civil Rights Data Collection Website. Suspensions and expulsions are being considered as intervening outcomes stemming from both the implementation of school safety tactics and policies and the occurrence of shootings as a way to understand the role of student criminalization.

#### Other publicly available data on schools and their neighborhoods

Other publicly available data specific to the study schools, their neighborhoods (measured as census tracts and block groups), and their states are also being collected specific to each year of the study period and linked to each school and their school safety and discipline outcome data. These independent covariates are being collected via multiple data sources at the NCES and via US Census data. They include key variables at the school level: political orientation (as measured by voting patterns data obtained from US Census Bureau’s county-level Voting and Registration estimates), policies mandated by state government and insurance regulators (e.g., schools designated as gun-free zones), urban/non-urban (determined using NCES locale codes), economic (determined using percentage of students in each school eligible for free and reduced price lunches as well as residents below the poverty threshold in the surrounding neighborhood), and race/ethnicity (measured as differences in each school as well as among residents in the surrounding neighborhood). Additional covariates specific to the proposed study’s schools and their surrounding neighborhoods will include school staffing characteristics, school budget, student academic performance, and the school’s physical plant (e.g., outdoor play areas, number of floors).

### Protection of human subjects

The large majority of data collected in this study do not involve human subjects. Indeed, much of the data collected in this study are publicly available. In the case that a school’s safety plan for a specific academic year of interest is not available online, the study directors are emailing the district’s superintendent of the school directly to inquire if they would be willing to provide a copy of the safety plan for the purpose of this research study. As a result, the superintendent of every participating school district in this proposed work is eligible for participation, but no identifying or private information about the superintendents themselves will be recorded.

Approval for this study was obtained from the Columbia University Human Research Protection Office and Institutional Review Board in November 2021 (IRB protocol number: AAAT9087). The study was deemed exempt from the requirement of obtaining written or oral consent.

### Data organization and management

#### Study design

The case-control study design is a natural choice in accomplishing the aims proposed here given the relatively rare outcome of school shootings and the need to quantitatively test school safety tactics and policies by including a national comparator group of control schools that have not experienced shootings. In addition, other study designs, such as randomized controlled trials or cohort studies, would be unethical to conduct or would take infeasibly long periods of time to complete, thus underscoring the benefits of utilizing the case-control study design as described here.

**Fig 1:**
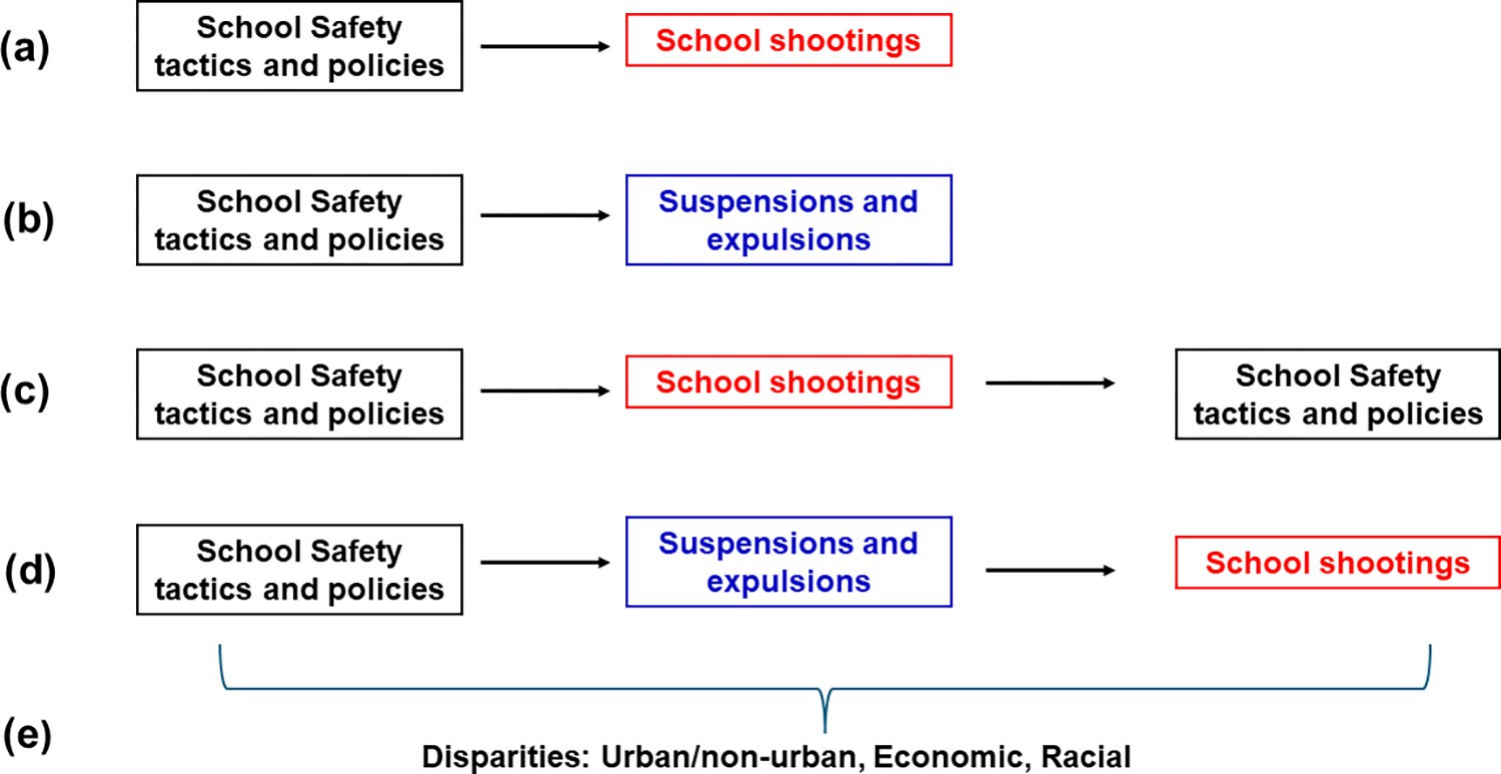
Conceptual framework and temporality of the proposed case-control study.

Per the study’s Specific Aims, this study is examining associations between safety tactics/policies and various outcomes (Fig 1). As described in Specific Aim 1, our research team is testing relationships between the total number and specific presence of 27 school safety tactics and policies using a case-control design to calculate the odds of intentional school shootings. In Specific Aim 2, we are testing the relationships between the total number and specific presence of 27 types of school safety tactics and policies and the occurrence of school suspensions and expulsions. Aims 1 and 2 are possible due to the temporal specificity of the data, as implementation of safety tactics/policies and occurrence of school shootings and suspensions/expulsions are date stamped.

In addition, our research team is exploring whether implementation of school safety tactics and policies prompts the occurrence of school suspensions and expulsions, which may then potentially lead to the occurrence of later school shootings. Some school shooters had been previously expelled or had a history of being suspended. Certain security tactics/policies may increase suspensions/expulsions, which can then affect school shootings. Aim 3 further delineates these potential relationships in terms of urban/non-urban, economic, and racial subgroups. This final aim is key to understanding how various relationships among safety strategies, suspensions/expulsions, and shootings play out for different populations and where school districts and policymakers may best and most effectively focus their safety efforts.

#### Data analysis plan and study registration

An estimated 658 K-12 public schools will be included in our study sample. The presence or absence (binary 0–1 variables) of each of the 27 safety tactics and policies in case and control schools before each case school’s shooting will be statistically tested as independent variables of interest. The number of safety tactics and policies that each school has in place (from 0–27) will be statistically tested as independent count variables. And the number of suspensions and expulsions that have occurred will also be statistically tested as a proportion of the number of students in each school. Specific safety tactics and policies found to have small numbers will also be combined into groupings of safety tactics and policies as per prior aggregated categories: external target hardening, internal target hardening, student/staff monitoring, emergency procedures/drills, emergency notification technologies, medical support, and school security staff.

Analyses will begin with descriptive statistics to characterize cases and controls relative to independent variables. Categorical and continuous variables will be explored using frequency distributions, means, medians, standard deviations, skewness coefficients, and ranges. Bivariable analyses of predictor variables for each outcome will consist of t -tests, Chi-squared tests, and nonparametric comparison tests. Collinear independent variables will be identified using Spearman coefficients, tolerance statistics, and condition indices. As this population-based case-control study will be matched on state, urban/non-urban, and elementary/middle/high school status, conditional logistic regression model specifications will be used. The first two aims will adjust for confounders that will be methodologically and theoretically justified. The third aim will include interaction analyses with select effect modifiers: urban/nonurban, economic, and racial disparities. Despite having matched on these variables, effect modification analyses with these variables are possible and commonly conducted in case-control studies. Disparities analyses will include adjustments for sparse data. Researchers will reasonably limit the final group of predictor variables to promote statistical efficiency and provide the best unbiased estimators.

Multiple testing in regression models will be handled by pre-specified variable selection procedures (Benjamini-Hochberg) to control false positive findings in terms of type I error rate and false discovery rate. Several regression models will be tested and compared using Wald statistics, and multiple testing adjustments and false discovery rates accounting for correlated conditions and dependency will also be used to provide appropriate estimates of statistical significance and statistical inference. Our research team has pair-matched to increase efficiency in a situation where the distributions of the matching covariates are drastically different in case and control populations. We will use a double robust, efficient, weighted targeted maximum likelihood procedure for the estimation of causal parameters in matched case-control study designs. Where missing data do exist and are missing at random, researchers will use multiple imputation.

Lastly, in line with best practices regarding data storage and in order to assure research quality and integrity, we will implement processes to maximize the rigor, reproducibility, and generalizability of our proposed study. Openness and transparency are being facilitated by pre-registering our planned methods, analyses, and covariates, and sharing of computer code, datasets, and research results. To reduce the likelihood of any possible publication or confirmation biases that might be skewed towards positive results, primary analyses and hypotheses involving tests of the school tactics and policies specified in our specific aims have been publicly pre-registered via clinicaltrials.gov. We will also take every care to ensure that relevant alternative arguments are considered when presenting our results. This will offer school districts and policymakers at the state and federal levels a sound evidence base for the development of fair, objective, and evidence-based policies.

## Discussion

Persistent school gun violence has led to schools adopting a number of school safety tactics and policies. Most of these school safety strategies are designed based on common knowledge rather than scientific evidence of their effectiveness. Despite their widespread implementation in public schools, the association between number and type of safety strategy with the occurrence of school shootings has not been sufficiently established through controlled scientific study. It is also unclear how these safety strategies are associated with school discipline outcomes such as suspensions and expulsions rates. Furthermore, it is not known how urban/non-urban, economic, and racial disparities modify the relationship between school safety tactics and policies, school suspensions and expulsions, and the occurrence of intentional gun violence at schools.

Implementation of school safety strategies also takes up resources in terms of finances and time from the school and therefore research on their effectiveness is required. At present there is no research that has documented the usefulness of the number and type of school safety strategies in preventing school gun violence. There is also a lack of evidence on how these strategies inform school discipline processes, and research is needed to establish if any association is present.

## Conclusion

The anticipated results of this work will fill a significant gap in our understanding of the effectiveness and functionality of various school safety tactics and policies in preventing school shootings, as well as their impact on student discipline. Ultimately, this study is intended to provide schools with data-driven evidence with which to choose, implement, and monitor safety tactics and strategies within a national sample of diverse school contexts and student populations, ultimately contributing to safer and more equitable learning conditions.

## Supporting information

S1 File Inclusion and exclusion criteria for case and control schools. (PDF)

S2 File School safety strategies and tactics. (PDF)
