# Supplementary material for: Protocol for a nationwide case-control study of firearm violence prevention tactics and policies in K-12 schools
Source: PLoS One. 2024 May 20;19(5):e0302622. doi: 10.1371/journal.pone.0302622 (PMC11104607; doi:10.1371/journal.pone.0302622)
Supplement: S1 File — (PDF) [file pone.0302622.s001.pdf]

Inclusion and Exclusion Criteria for case and control schools

Case schools

Inclusion criteria

For a case school to be included, it must satisfy the following 2 criteria:

1. *The incident must have taken place on the school property.*
    a. This includes - all school premises including school parking lots, playgrounds, fields, tracks, etc.
        i. Front sidewalk or walkway immediately adjacent to school is also included
    b. Because school buses do not meet this criterion, all incidents on a school bus will be excluded *unless the bus is parked in the school.*
    c. Off-campus school events that take place at other locations such as a church, a community center, or other type of location are also not included for this reason.
    d. One exception to this rule is when the shot is fired outside of the school property, but the bullet ends up hitting someone/something on the school property.
2. *The gun must have been shot during school hours.*
    a. School hours also include one hour before and after the official hours, keeping in mind the arrival and dismissal activities.
        i. We have estimated this typically covers the period between 6AM – 6PM (recognizing that many schools begin at 7AM and end after school activities at 5PM). However, the exact time of each incident will be checked alongside the school's hours to ensure it meets this criterion.

b. This also includes any school sanctioned events happening after hours on the school campus - such as football games, school dances, and other similar events. (The rule of one hour before/after the event is applicable here too).

c. Students/faculty/staff- anyone required on the campus will be considered as school being in session.

    i. While schools were closed due to COVID-19, some staff and teachers were coming to campus to take online classes. If a shooting happens in such a case, it will be included.

Important Notes:

- Gunshot: Gunshot *must* be from a powder-discharge firearm. BB guns, pellet guns, stun guns, etc. are NOT included.
- Shooter: The location of the shooter does NOT matter. Shooter can be off-campus.

The following characteristics do NOT influence whether we include the incident as a case:

- The number of people shot/injured. We are interested in all intentional school shootings, even if no one was physically hit with a bullet.
- Relation of the shooter to the school - could directly, indirectly, or not related to school in any way. We are interested in all intentional shooting incidents, regardless of the shooter's connection to the school
- We are including all incidents that meet our above criteria, regardless of whether specific school property was hit. (For example, an incident where a shooter fires a gun into the air outside in the parking lot, it would count if it met our other criteria).
- The motivation for the incident doesn't matter, as long as it is an intentional shooting meeting our criteria above.

Exclusion Criteria

A school will be excluded from the final list if

1. It doesn't meet either one or both inclusion criteria described above.
2. The shot was not intentional.
3. If it was a suicide/attempted suicide and there was no attempt to shoot another person.
    a. Murder-suicides will be included.
4. The shot was fired on the school bus on its way to or from the school or another school-sanctioned event. The bus was not parked at the school.
5. The school was not in session for any reason (e.g. closed for a holiday, COVID-19, etc.).
6. The school event was taking place at a location where school security measures were ineffective, that is off the school property.
7. The shot originated from outside the school and no person or property was hit in the school. If the school went into a precautionary lockdown after hearing the shot(s), the incident still doesn't meet the inclusion criteria based on this.
8. If the SRO/police responding to an event was the only person to discharge a weapon.

Summary of rationale for each criterion

**Criteria**	**Decision**	**Rationale**
Gun is fired on the school property	Likely Include	Given that we are studying school security measures against active school shootings, any shooting that happened on campus has the potential to be included.

Gun is fired outside of the school property but someone/something at school is hit	Likely include	Even though the shot originated off campus, there are incidents where the shot hits someone or something at the school. In this case, the incident would be included.
Gun is fired during school hours/activities	Likely include	All shootings that take place during school hours and activities are included (see note above re: specificity on timing).
School sanctioned events on the school campus	Likely include	Include because when schools sanction an event, the school's typical security measures should be in place.
Suicide-murder	Likely include	Given that person has an intention to hurt other(s)- this shall be included
Gun is fired outside of the school property and no school person/property was hit	Likely exclude	Since the school security measures can't be extended to off campus locations. (A school lockdown in such a case is considered precautionary).
Gun fired on a school bus while off campus	Likely exclude	Since the school security measures can't be extended to school buses, such incidents will be excluded.
Gun is fired after school hours/activities	Likely exclude	Incidents that take place when the school building is closed (e.g. at night when there are no students or staff in the school and no after hour school events happening) will not be included. (It is unlikely that most (if any) security measures can be implemented in response to these incidents).
School sanctioned events off-campus	Likely exclude	It is unlikely that the school's security measures can be extended to off campus spaces, even if this is a school sanctioned event
Number of people shot/injured	No effect on inclusion/exclusion	The shooter could have missed the shot and doesn't mean the next shot would not have hurt someone.

Relation of the person shot at school	No effect on inclusion/exclusion	Because a person could have shot more than one person- and this incident will need security measures activated
Property damage	No effect on inclusion/exclusion	The shooter could have missed the shot, or if fired in air may have the intention to shoot a person next
Intention	No effect on inclusion/exclusion	The intention of a person to shoot a student/staff/parent isn't a deterrent to other people getting hurt. Things can escalate and security should be in place.

Control Schools

One control school for each case school will be randomly selected from *the US Department of Education's national databases of all K–12 public schools* based on the following criteria:

Inclusion criteria

1. Controls will be selected from a national database of all K-12 public schools.
2. The control school must NOT have experienced an intentional shooting during the 5 years prior to the start of the present study (1/1/2015); that is 1/1/2010 onwards.
    - Note: The control school that experienced a shooting incident* after the matching case school is still eligible. *(Also see exclusion criteria 3)*
3. Control school must match the corresponding case school on the following three criteria-
    I. US state of the case school
    II. Urban versus non-urban status of the case school
    III. Case school's status as elementary/middle/high school

4. The school was operating, and safety plans are available for the academic year of interest for the corresponding school. *(Also see Missing data)*

Exclusion Criteria

1. All non-public schools (e.g., private, charter, parochial schools) and schools outside of the K-12 range (e.g. daycares, preschools, and universities) are not eligible for random selection.
2. All schools in the same school district as the case school will not be eligible for pairing with that case school. Pair-matched case-control schools must be in separate school districts.
3. Any school that experiences an intentional school shooting will be subsequently excluded from the random selection for the remaining study period.
   a. Note: A school can be in both case and control school lists, however, once a control school has experienced an intentional shooting it is no longer eligible for random selection as control school in this study.

Missing Data

If it is not possible to obtain the required data for the control school, the reason for the school's missing data will be documented (see list below for likely causes of missing data) and the control school will be replaced with the next school on our list that meets all the above criteria-

1. If the randomly selected pair matched control school wasn't operating for the academic year of interest for the case school.
2. If the randomly selected control school is no longer in operation and no one can be reached for retrieving its safety plans.
3. If the matched control school's safety plans are not available for any other reason.
4. If the control school declines to participate in the study.
