# Supplementary material for: Protocol for a nationwide case-control study of firearm violence prevention tactics and policies in K-12 schools
Source: PLoS One. 2024 May 20;19(5):e0302622. doi: 10.1371/journal.pone.0302622 (PMC11104607; doi:10.1371/journal.pone.0302622)
Supplement: S2 File — (PDF) [file pone.0302622.s002.pdf]

# School Safety Strategies and Tactics

| **Category** | **School Safety Strategy or Tactic** |
|---|---|
| External target hardening | Locked, monitored, and illuminated school entry doors and/or other external lights |
| | External barriers (including window bars), bollards, and buffer zones |
| | Having an “open door” or “open campus” policy |
| | Sign(s) indicating the school is a “Gun Free School Zone” |
| Internal target hardening | Metal detectors |
| | Classroom doors that lock from the inside |
| | “Wing walls” or curved hallways |
| | Bulletproof structures |
| | Security cameras |
| Student/staff monitoring | Threat assessment team |
| | Conducting random sweeps for contraband |
| | Requiring clear book bags or banning bag bags |
| | Requiring students and/or staff to wear identification |
| | Zero-tolerance policies for weapons, drugs, acts/threats of violence |
| Emergency procedures/drills | Written active shooter plan |

| | |
|---|---|
| | Written hostage situation plan |
| | Written bomb threat plan |
| | Written suicide threat plan |
| | Implementation of lockdown or active-shooter drills |
| Emergency notification technologies | Automated parental notification in a school-wide emergency |
| | Panic buttons that directly connect to police |
| | Anonymous threat reporting system (e.g. a “tip line”) |
| Medical support | Full or part-time school nurse on school campus |
| | Full or part-time school psychologist or other mental health counselor/staff (e.g. licensed social worker) on school campus . |
| | First-aid training for students and/or school staff |
| School security staff | Non-police school security or resource officers |
| | Law enforcement officers or police |
| | Teachers and/or other personnel armed with guns at school |
